# Routine Fundoscopy: Unravelling Undiagnosed Significant Coarctation of the Aorta

**DOI:** 10.7759/cureus.76075

**Published:** 2024-12-20

**Authors:** Sripathi Kamath, Akshata Charlotte, Pooja Peethambaran, Madhurima Nayak, Sunayana Bhat

**Affiliations:** 1 Ophthalmology, Father Muller Medical College, Mangalore, IND

**Keywords:** arteriolar tortuosity, coa (coarctation of the aorta), disk edema, fundoscopy, hypertensive retinopathy, pediatric hypertension, retina hemorrhage

## Abstract

A 10-year-old boy was brought to the outpatient department with complaints of diminished vision for two years. On examination, the best-corrected visual acuity (BCVA) in oculus dexter (OD) was 20/40 and in oculus sinister (OS) was 20/80. The patient was dilated for routine fundoscopy, which revealed grade IV hypertensive retinopathy changes in both eyes, with a macular fan in the left eye. The patient’s blood pressure was promptly checked and, as suspected, was high. An emergency pediatric medicine consultation was done where it was noted that the patient’s peripheral pulses were feeble, and he had cold extremities. The blood pressure was 200/140 mmHg in the upper limb and 90/60 mmHg in the lower limb. An echocardiogram revealed significant coarctation of the aorta (CoA), further confirmed by a CT angiogram. This case report highlights the importance of comprehensive fundoscopic examination for detecting potentially fatal cardiac conditions.

## Introduction

Hypertension in children, though less common than in adults, can often indicate underlying systemic or congenital conditions. Among these, coarctation of the aorta (CoA) is a significant cause of pediatric secondary hypertension, accounting for 5%-8% of all congenital heart defects. Early detection and timely intervention are critical to improving long-term outcomes in such cases. However, CoA often presents with subtle signs, leading to delayed diagnosis and treatment.

The retina, as the only site in the body where the microvasculature can be directly observed, plays a crucial role in detecting hypertensive changes [1]. Fundoscopy has long been recognized as an essential tool for diagnosing hypertensive retinopathy, which can serve as a window to systemic vascular health. While characteristic retinal features such as corkscrewing of arterioles are common in CoA, these may not always be present, especially in younger patients or those without long-standing hypertension.

This case report highlights the pivotal role of a routine ophthalmologic examination in uncovering an undiagnosed case of CoA in a 10-year-old boy. By presenting an atypical presentation of hypertensive retinopathy without the classic retinal arteriolar tortuosity, this case underscores the importance of a thorough fundoscopic evaluation and its potential to reveal life-threatening systemic conditions.

## Case presentation

**Figure 1:**
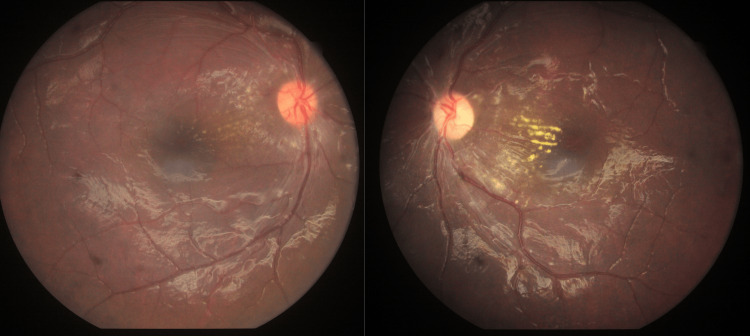
Fundoscopic images depicting retinal changes with disc edema

**Figure 2:**
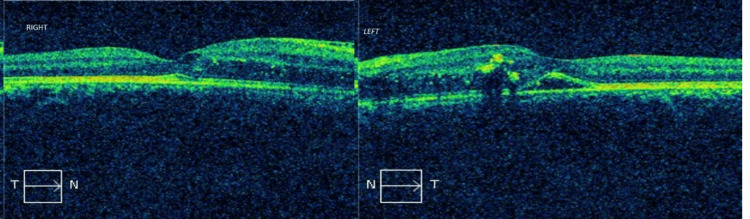
Optical coherence tomography (OCT) imaging demonstrating disc edema in both eyes and left neurosensory retinal detachment

A 10-year-old boy presented to the ophthalmology outpatient department (OPD) for refractive error assessment. He had been using spectacles for two years and had discontinued them for the last one year. Additionally, he complained of intermittent headaches for the past four years. Headache was predominantly in the right frontal region, occurring once every three to four months and often associated with nausea and vomiting. Additionally, the patient reported vision abnormalities over the past two years, characterized by difficulty focusing on both near and far. There was no history of photophobia, trauma, fever, or seizures. On examination, there was 15° concomitant esotropia. His unaided visual acuity was 20/60 in the right eye and 20/120 in the left eye, and his best-corrected visual acuity (BCVA) was 20/40 and 20/120 in the right and left eyes, respectively. As a part of his evaluation, intraocular pressure was checked and was found to be normal. Anterior segment examination of both eyes was unremarkable. Fundus examination of both eyes revealed disc swelling with generalized narrowing of arterioles. The veins were normal and did not exhibit tortuosity. Hard exudates were also seen arranged in the form of a fan nasal to the macula. Figure 1 shows the fundus examination findings. Optical coherence tomography (OCT) macula of both eyes showed nasal macular edema with left eye neurosensory retinal detachment. Figure 2 shows the OCT findings.

**Figure 3:**
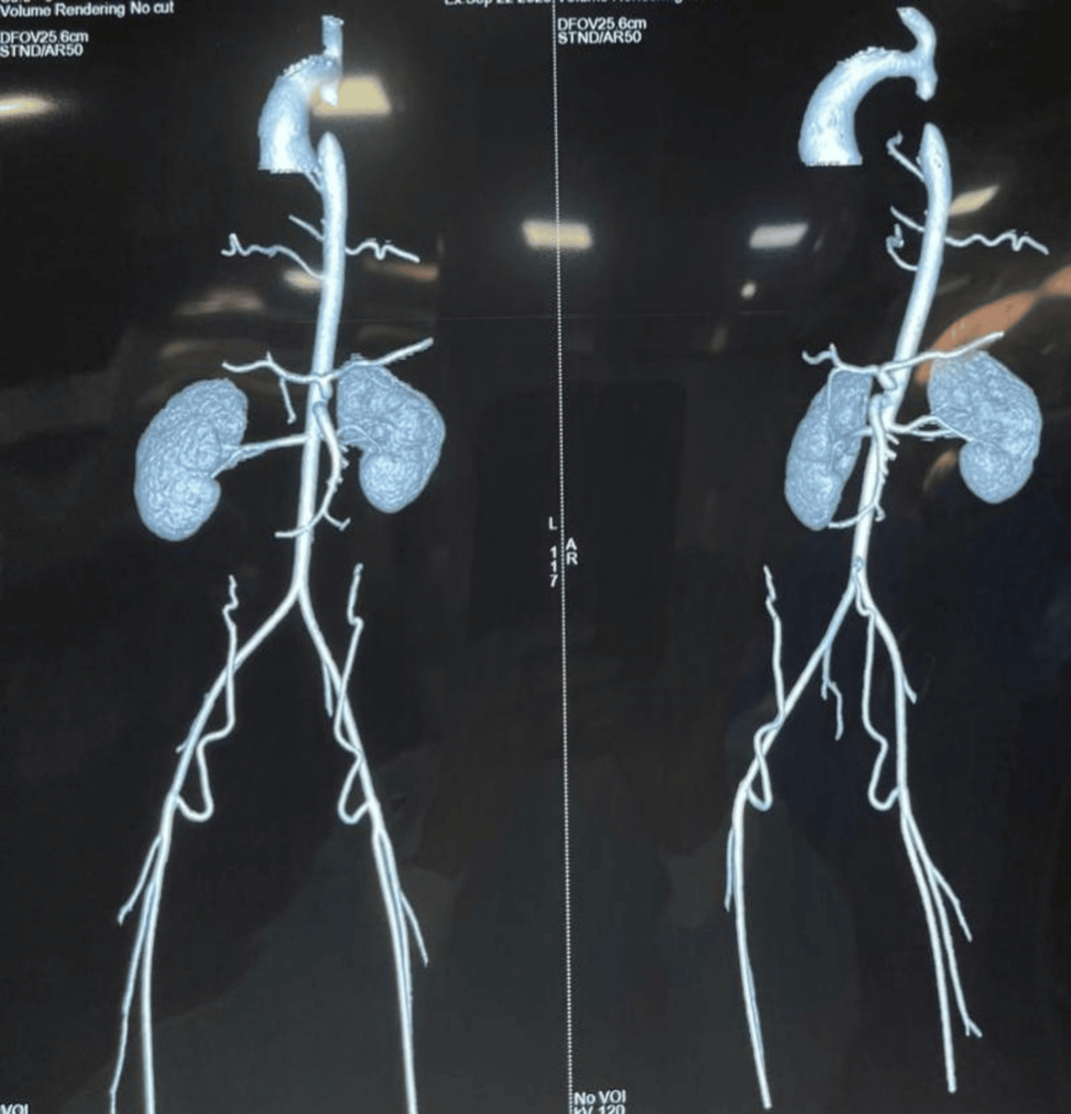
CT angiography of the aorta demonstrating severe coarctation of the aorta distal to the left subclavian artery

Blood pressure showed significant variations in the upper limb (220/140 mmHg) and lower limb (90/60 mmHg). The patient had feeble pulses and cold extremities. Further evaluation using a 2D echocardiogram revealed significant CoA. This finding was further confirmed by CT angiography, which showed occlusion in the descending aorta, 15 mm below the origin of the left subclavian artery, likely aortic coarctation, which had progressed to complete obstruction with extensive collateral vessels. Figure 3 shows CT angiography suggestive of CoA.

**Figure 4:**
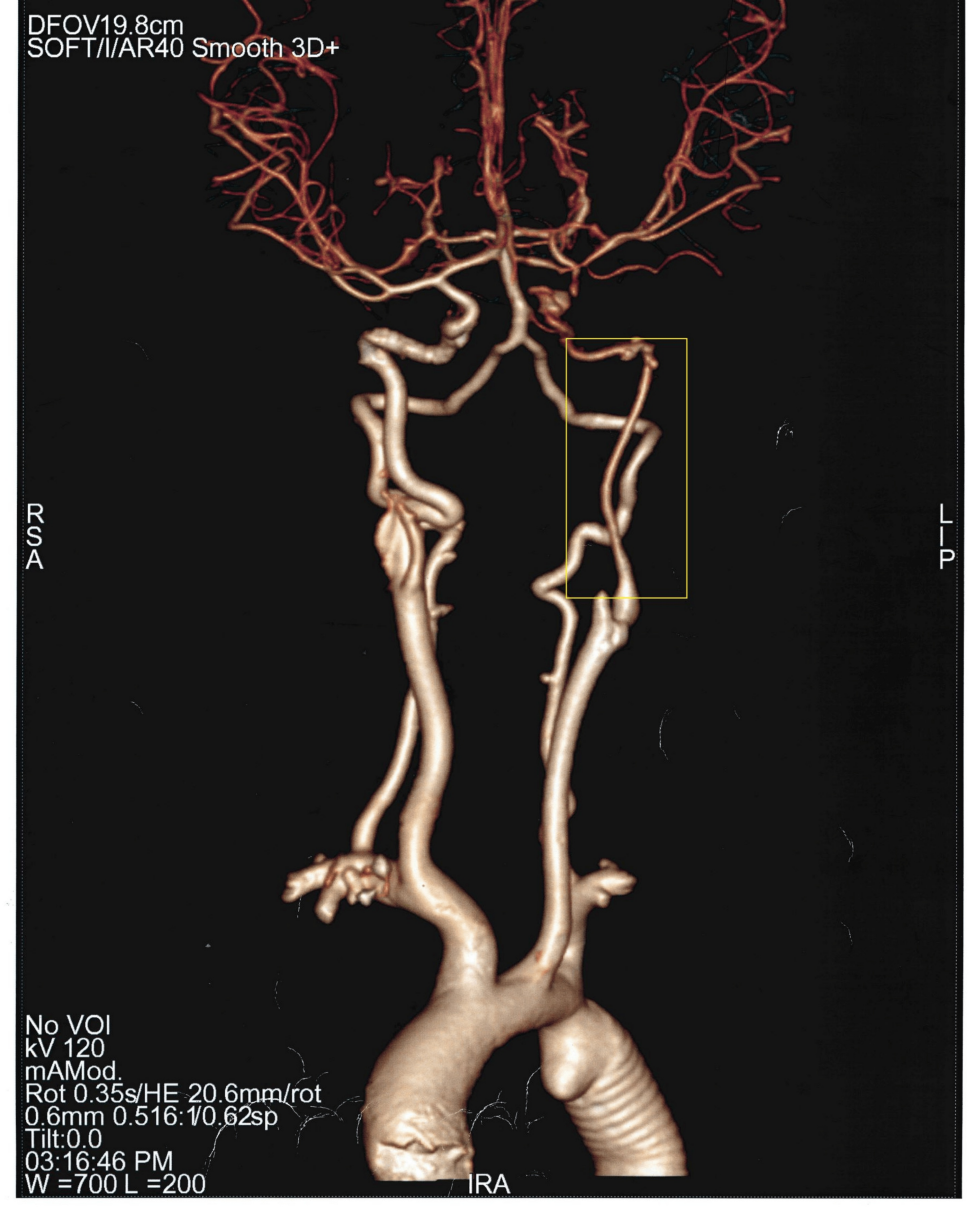
3D reconstruction of the aorta showing graft in situ following coarctation repair

Based on all these findings, the patient was diagnosed with significant CoA with grade II mitral regurgitation. A pre-operative evaluation was done, and he was taken up for coarctation repair with a 16-mm dacron graft to the distal aorta. Figure 4 shows post-procedure CT angiography showing the graft in situ. At the time of discharge, his vital signs were stable, with blood pressure and heart rate within the target range due to the antihypertensive therapy initiated. The surgical site was healing well, and no immediate post-operative complications were noted. He was kept on a monthly follow-up and is currently doing well.

## Discussion

A 10-year-old boy presented to the ophthalmology OPD with a history of diminished vision and intermittent headaches. Routine dilated fundoscopy revealed grade IV hypertensive retinopathy, including disc swelling, narrowed arterioles, and macular edema with a fan-like formation in the left eye. Upon further workup, his blood pressure was markedly elevated in the upper limbs compared to the lower limbs, with feeble peripheral pulses and cold extremities. Subsequent echocardiography and CT angiography confirmed significant CoA with a complete obstruction in the descending aorta and extensive collateral formation. We report a case of CoA that was diagnosed due to the presence of grade IV hypertensive retinopathy in a 10-year-old unsuspected male patient who presented for refractive error correction. This case underscores the role of comprehensive ophthalmic evaluation in identifying rare systemic conditions like CoA.

The retina holds a unique position in which we can directly visualize the microvascular structure, which provides a preview for the detection of arterial hypertension or cardiovascular disease. Numerous vascular conditions can be diagnosed by close observation of the retinal vasculature. One example of this is the CoA. CoA is characterized by partial or complete stenosis of the aorta near the insertion site of the ductus arteriosus, causing high blood pressure above the site of obstruction and relatively normal or low pressure below it. As a result, changes in the retinal arterioles contribute to certain signs of hypertensive retinopathy. However, the hypertensive changes in CoA are different from those due to juvenile hypertension and are mainly distinguished by corkscrewing of retinal arterioles [1].

Secondary hypertension is a term used to describe hypertension resulting from other illnesses. The majority of etiologic causes of secondary hypertension in children are cardiovascular, endocrine, and renal-vascular disorders. Primary or essential hypertension is a term used to describe hypertension in cases where a secondary cause cannot be identified. A multitude of factors, including stress, nutrition, obesity, and heredity, contribute to the development of essential hypertension. In children, essential hypertension is quite uncommon [2].

CoA is one of the important causes of childhood hypertension [3]. Clinical features include absence or diminished femoral pulses, low leg pressure relative to arm pressure, and changes in heart size, rate, rhythm, and murmur [4].

Schwartz reported five cases of CoA, wherein tortuosity and sclerosis of retinal arterioles were present in four cases [5]. Retinal artery tortuosity has been documented in 70% of patients with CoA [6], while both arterioles and veins are typically affected to a similar degree in other causes of juvenile hypertension. In cases of CoA, tortuosity is primarily limited to the arterioles.

In CoA, the upper half of the body frequently has “corkscrew-shaped“ arterial tortuosity, which becomes more noticeable as one ages [1]. This typical form of tortuosity involves side-to-side vessel bends that are primarily restricted to a single plane [6]. Undoubtedly, pliable vessels, high systolic blood pressure, relatively wide pulse pressure, and decreased peripheral resistance are significant factors contributing to the development of tortuosity in coarctation. These factors tend to intensify as the disease progresses [7]. However, in our case, we did not observe retinal arteriolar tortuosity most probably because of the young age of the patient: the hypertension would not have been long-standing enough to cause the above-said changes.

Systemic artery health can also be ascertained through examination of the retinal arteries. In the presence of malignant hypertension, other findings such as hemorrhages, cotton wool spots, and disc edema are observed. Retinal arterioles are attenuated, but not tortuous, in cases of hypertension resulting from other causes [8].

Since the patient was young, there were very subtle tell-tale signs pointing toward a detailed systemic evaluation. Mild disc edema with attenuated arterioles and old retinal hemorrhages with a nasal macular fan pointed to grade IV hypertensive retinopathy as a diagnosis. Despite the well-established fact that corkscrewing of retinal arterioles occurs in 70% of instances with CoA, this patient did not exhibit it. An intriguing finding was the absence of concomitant nephropathy or renal parenchymal disease, which is the most frequent cause of malignant hypertension. These are taken together to add to the singularity of this case. 2D echocardiogram showed significant CoA. This is most likely the result of aortic coarctation, which has advanced to full obstruction, involving extensive collateral arteries. This demonstrates how crucial fundoscopy is, for aiding in the diagnosis of systemic illnesses.

In this case, the patient presented with signs of grade IV hypertensive retinopathy, as evidenced by mild disc edema, attenuated arterioles, old retinal hemorrhages, and a nasal macular fan. These findings are characteristic of malignant hypertension but do not specifically point to CoA. In typical cases of CoA, we expect to see certain distinct retinal features, especially tortuosity or “corkscrewing” of the retinal arterioles, which was notably absent in this patient. This absence of CoA-specific retinal changes is unusual, as studies report that up to 70% of CoA patients exhibit this corkscrewing appearance in the retinal arterioles, likely as a result of chronic hypertensive load proximal to the narrowed aortic segment.

The absence of tortuosity in this case suggests an atypical retinal response to hypertension caused by CoA, highlighting a unique pattern. Retinal arteriolar attenuation, a hallmark of hypertensive retinopathy in general, was present instead, aligning with systemic hypertension rather than specifically CoA-induced hypertension. The presence of old hemorrhages and a nasal macular fan further underscores this alignment with classic malignant hypertension findings, which commonly show such retinal changes, particularly in high-grade retinopathy.

Interestingly, no renal involvement was identified, which typically serves as the primary cause of malignant hypertension [9]. This lack of nephropathy further singles out this case, directing suspicion toward CoA. However, the absence of tortuous retinal arterioles despite significant CoA suggests that other compensatory mechanisms or collateral circulations might have mitigated the impact on the retinal microvasculature. Indeed, the 2D echocardiogram findings confirmed an extensive collateral network around the site of obstruction, possibly reducing the direct pressure impact on the retinal vessels that typically leads to corkscrewing.

## Conclusions

This case underscores the value of a detailed fundoscopic examination, as the hypertensive retinopathy findings ultimately pointed to the need for systemic evaluation. While the retinal changes were more indicative of generalized hypertensive retinopathy than CoA-specific changes, they were instrumental in revealing a severe underlying systemic condition. This case exemplifies how retinopathy findings can vary, even within conditions where specific retinal features are expected, and the importance of keeping a broad differential when interpreting these signs.
